# Editorial: Best practice approaches for mixed methods research in psychological science, volume II

**DOI:** 10.3389/fpsyg.2025.1647507

**Published:** 2025-09-18

**Authors:** Gudberg K. Jonsson, Mariona Portell, José Luis Losada, Judith Schoonenboom

**Affiliations:** ^1^Social Science Research Institute and Human Behavior Laboratory, University of Iceland, Reykjavík, Iceland; ^2^Department of Psychobiology and Methodology of Health Sciences, Universitat Autònoma de Barcelona, Cerdanyola del Vallès, Barcelona, Spain; ^3^Department of Social Psychology and Quantitative Psychology, Universitat de Barcelona, Barcelona, Spain; ^4^Department of Education, University of Vienna, Vienna, Austria

**Keywords:** symmetry, quantitizing, mixed methods, record transformation, Qual–Quan integration, complexity

## Introduction

The evolution of mixed methods research in psychological science marks a pivotal shift from the historically entrenched divide between qualitative and quantitative paradigms. Mixed methods emerged in the late twentieth century as a reconciliatory approach. By integrating diverse methodologies, it enriched psychological inquiry and gained momentum. Over the past decades, its adoption has expanded exponentially across disciplines, reflecting its versatility in addressing complex research questions. Yet, this growth has also introduced challenges, including conceptual ambiguities, heterogeneous design taxonomies, and inconsistent quality in application. As the field matures, establishing methodological rigor and standardized criteria remains critical to ensure the credibility and coherence of mixed methods research.

The way psychological and educational research is conducted has changed significantly over the last two decades. Researchers are increasingly working across disciplines and internationally, and new technologies have enabled them to develop new methods. As a result of these two tendencies, research projects and research questions have become much more complex and dynamic. It has become increasingly necessary to use more than one method to answer these questions, leading to a steady increase in mixed methods studies. Moreover, mixed methods research itself has become more complex, interdisciplinary, and collaborative. Previously, the basic idea of mixed methods research involved a combination of qualitative and quantitative data collection, analyzed separately, and then brought together by a single researcher or a small team at the end of the study. However, this is no longer the case.

*Volume II of Best practice approaches for mixed methods research in psychological science* reflects this increase of these diverse forms of complexity. It builds on the highly successful inaugural volume of “*Best practice approaches for mixed methods research in psychological science*” (Anguera et al., 2020). We will discuss the relation between the two further below.

## Developments in mixed methods research with different forms of data collection

The complexity of mixed methods research grows as more studies are conducted by interdisciplinary teams. Poth et al. champion integrative teamwork in mixed methods research, proposing strategies to harness disciplinary synergies. Their reflection on a literacy case study identifies enablers (e.g., shared frameworks) and barriers (e.g., methodological silos), advocating for collaborative capacity-building to tackle complex problems.

As psychological research moves away from performing cross-sectional research to develop theories about change and toward longitudinal research, complexity increases, but so do opportunities for mixed methods research. Lykkegaard and Qvortrup track tertiary students' dropout considerations, revealing fluctuating motivations shaped by academic-personal alignment. Their mixed methods design captures both stable trends and individual variability, challenging linear assumptions about dropout pathways.

As for tracking data, the COVID pandemic brought new challenges as well as new digital interventions, including Corona apps. Fiol-deRoque et al. evaluated PsyCovidApp, a digital tool for healthcare workers. A questionnaire on the app's use and perceived utility was enhanced by interviews with users that explored these topics in greater depth and identified barriers and suggestions for improvement. The latter topic was the subject of additional panels with experts. As a result, not only could the most important benefits and barriers be identified, but also suggestions for optimizing the process could be generated.

An innovative method combination involving questionnaire data is described by Buchholtz and Vollstedt. As in the two previous studies, Buchholtz and Vollstedt use interview data to study their questionnaire outcomes in depth, but they do so by utilizing an innovative intermediate step: interviewees were asked to Q-sort their questionnaire responses, which were analyzed quantitatively to develop different profiles.

## Quantitization: building profiles and patterns from quantitized qualitative data

The articles discussed above involved innovative mixed methods studies that analyzed separate quantitative and qualitative data with the aim of understanding the process behind quantitative results, through the analysis of qualitative data separately collected, in this case interviews.

The majority of articles in this Research Topic follow a different pattern. These articles employ a methodology in which qualitative data are quantitized. In contrast to the previous articles, these articles start with qualitative data, which is coded in great detail. These codes are then counted, and correlational and temporal patterns are used to develop patterns and profiles. In these articles, there is only one round of data collection, namely qualitative data collection, which is subsequently quantitized and interpreted qualitatively. In mixed methods notation, the design of this methodology is QUAL → QUAN → QUAL (Anguera et al., 2020). Since there is only one round of data collection, these articles do not combine two methodologies. Instead, they rely on a single integrated methodology that takes qualitative data as input, quantifies it using detailed coding, and then interprets it qualitatively.

Although the pattern QUAL → QUAN → QUAL applies to all studies using this methodology, the collection methods, types of coding and patterns derived can vary hugely. This was already visible in the inaugural volume of “*Best practice approaches for mixed methods research in psychological science*” (Anguera et al., 2020), which comprised 32 manuscripts that advanced both methodological innovations and their applications across disciplines, of these 19 manuscripts that used this methodology. This Research Topic introduced technological breakthroughs in data collection methods, including sensor-based approaches and specialized analytical software such as LINCE (Soto-Fernández et al., 2022), SAGT (Hernández-Mendo et al., 2016), GSEQ (Bakeman and Quera, 2001), HOISAN (Hernández-Mendo et al., 2012), and THEME (Magnusson, 1996, 2020; Magnusson et al., 2016). Many contributors adopted a mixed methods approach, drawing on the “connecting” notion (Creswell and Plano Clark, 2007) and on subsequent methodological developments of this same logic proposed by Anguera et al. (2017, 2018, 2021). The methodological approaches featured T-Pattern Analysis, polar coordinate analysis, and sequential analysis applied across domains spanning sports, education, clinical psychology, and conversation analysis, demonstrating the breadth of mixed methods utility in psychological inquiry.

The foundation of this methodology, quantitization, is described by Onwuegbuzie. Onwuegbuzie reimagines quantitizing—the transformation of qualitative data into quantitative forms—via the DIME-Driven Model (Descriptive, Inferential, Measurement, Exploratory). This meta-framework addresses philosophical and practical barriers to quantitizing, advocating for its strategic use to enrich analysis without compromising qualitative depth.

Using the QUAL → QUAN → QUAL methodology to build patterns and profiles, various articles in this Topic advance foundational techniques for integrating qualitative and quantitative data. Belza et al. delve into a necessary methodological step to identify key aspects of the choreography followed by Pikler educators during breakfast, with the aim of developing a targeted training plan. Molinero et al. propose a therapeutic communication laboratory leveraging digital tools (e.g., ELAN, THEME) to analyze psychotherapy sessions through a Qual–Quan–Qual framework. Their work highlights how automated transcription and labeling enhances collaboration between practitioners and researchers, bridging theory and practice.

Similarly, Hunyadi explores eye tracking paired with T-pattern analysis to detect grammatical violations, revealing how behavioral patterns (e.g., gaze duration) correlate with syntactic processing. While initial hypotheses about total gaze duration were unsupported, the study suggests combining eye tracking with neurophysiological methods (e.g., ERP: Event-Related Potentials) for richer insights. Finally, Chacón-Moscoso et al. present a case study on workplace climate assessment, systematizing interview data through quantitizing and polar coordinate analysis. Their approach demonstrates how mixed methods can unveil hidden dynamics in organizational settings, offering actionable strategies for improvement.

## Quantitization: applications across contexts

Using the QUAL → QUAN → QUAL methodology to build patterns and profiles, various contributions illustrate mixed methods' versatility in addressing real-world challenges. In education, Qiao et al. identify group metacognition as a critical driver of collaborative learning outcomes, revealing three performance categories (H_T, EF, L_T) tied to metacognitive interactions. Their findings advocate pedagogical strategies that foster collective reflection. Tronchoni et al. analyse participatory interaction in university lectures using lag sequential analysis, uncovering 12 dialogical patterns that promote deep learning. Their work underscores the value of non-intrusive observation in refining instructional practices.

Bonilla Rodrìguez et al. investigated conflict management strategies in secondary school classrooms using a mixed methods design based on indirect observation, following the QUAL → QUAN → QUAL framework, on teachers' focus groups. The findings revealed notable gaps in teachers' understanding of conflict dynamics and highlighted a reliance on intuitive rather than systematically trained strategies. The study advocates for enhancing teachers' cognitive and emotional skills to improve classroom conflict management and adaptive coping mechanisms. Alarcón-Espinoza et al. applies a similar procedure to analyze interactions between teachers and students, demonstrating how the detected diachronic regularities contribute to understanding emotional regulation and classroom climate in daily educational settings.

Gläser-Zikuda et al. examine reflective writing through concurrent MMR, combining linguistic analysis and content analysis. Their work sheds light on how levels of reflection can be both qualitatively and quantitatively assessed, offering insights into teacher education and pedagogical growth.

Cultural and clinical applications feature prominently. El Khayat et al. employ polar coordinate analysis to study Pakistani mothers in Catalonia, revealing how migration sustains traditional parenting values despite acculturation pressures. Meanwhile, Santisteban et al. decode motor behavior in piano performance through observational methodology, linking tactile techniques (e.g., pressed vs. struck touch) to pedagogical strategies.

Farina and Pepe correlate adolescents' metaphorical narratives with wellbeing during COVID-19, showing how alexithymia exacerbates emotional confusion—a finding with implications for youth mental health interventions.

## Systematic reviews and perspectives: synthesizing knowledge

The QUAL → QUAN → QUAL methodology can also be useful in systematic reviews. Whereas, systematic reviews commonly rely on the researchers coding articles on specific themes, the Systematic Review by Kim and Cruz synthesizes 150 studies on PE teachers' leadership using text mining and meta-analysis. Their review highlights autonomy-supporting behaviors as key to student motivation and engagement. Regional disparities in research focus (e.g., health in Asian/European studies) call for culturally tailored interventions.

## Emerging themes and future directions

Three cross-cutting themes emerge:

- Integration as Innovation: Techniques like polar coordinate analysis, lag sequential analysis, and Q methodology demonstrate how blending qualitative depth with quantitative precision yields novel insights.
- Technology as Catalyst: Digital tools (e.g., LINCE, THEME) and automated processes enhance methodological rigor while democratizing access to mixed methods approaches.
- Contextual Sensitivity: From migrant parenting to pandemic-era mental health, studies emphasize the need for culturally and contextually adaptive frameworks.

## Conclusion

This Research Topic exemplifies the transformative potential of mixed methods in psychological science. Building on the foundation laid by Volume I, this second volume addresses persistent methodological challenges through carefully selected studies that exemplify genuine integration of qualitative and quantitative approaches. The included studies transcend traditional methodological boundaries, advancing both theory and practice by emphasizing conceptual innovations, methodological advancements, and practical implementations across education, mental health, cultural adaptation, and organizational behavior.

Collectively, these works illuminate complex psychological phenomena, offering insights unattainable through single-method approaches while providing researchers with clear pathways for navigating mixed methods design. They offer scalable solutions for education, healthcare, and beyond.

As the field evolves, fostering methodological literacy and interdisciplinary dialogue remains paramount. The contributions herein not only reflect the current state of the art but also chart a course for future research that is integrative, context-sensitive, and technologically empowered.

## In memoriam

We dedicate this manuscript to our dear friend and coauthor, Prof. M. Teresa Anguera, who passed away on January 19th, 2025. Dr. Anguera was a pioneering force in observational methodology and program evaluation design. Her groundbreaking work led numerous competitive research projects and was widely published in leading international journals. Her innovative integration of qualitative and quantitative approaches helped shape the field of Mixed Methods research.

As founder and first coordinator of the research group GRID (Research and Innovation in Designs: Technology and Multimedia Applications to Observational Designs), she championed the application of multimedia and digital technologies in behavioral research. In addition to this group, recognized by AGAUR (Agency for Management of University and Research Grants, Generalitat de Catalunya, Spain), she also led a much larger group that gained recognition through the awarding of multiple research projects in competitive calls. Her efforts enabled highly precise coding and analysis of complex human behaviors. She was also a founding member of the research group MASI (Methodology for the Analysis of Social Interaction), established at the University René Descartes, Paris V, Sorbonne, in 1995. Originally created through a partnership among six European universities, MASI has since grown into an international network with over 40 member institutions across Europe and the Americas.

Dr. Anguera's work consistently bridged theory and practice, providing rigorous methodologies for the study of social interaction and human dynamics. At the University of Barcelona, she taught at all academic levels and contributed significantly to graduate programs in Spain and Portugal, where she was widely admired for her clarity, rigor, and generosity as an educator. Her distinguished career earned her multiple honorary doctorates in recognition of her lasting academic impact.

Yet beyond her scholarly accomplishments, Teresa was deeply admired for her humility, ethical integrity, and unwavering commitment to mentorship. She inspired generations of researchers to pursue science with both intellectual precision and human empathy. An online interview, conducted by the Spanish Academy of Psychology, of which Teresa was a full member, allows us to preserve her voice and legacy, providing valuable insights into her vision and contributions to the field.

We mourn the loss of an exceptional colleague, mentor, and friend. Her wisdom, kindness, and dedication left a lasting imprint on the research community. Teresa's legacy lives on through the methodologies she developed, the collaborations she fostered, and the many lives she touched. Her memory will remain a guiding light for all who had the privilege of knowing her.

## References

[B1] Anguera M. T. Blanco-Villaseñor A. Jonsson G. K. Losada J. L. Portell M. (2020). Editorial: best practice approaches for mixed methods research in psychological science. Front. Psychol. 11:590131. 10.3389/fpsyg.2020.590131 33424707 PMC7793979

[B2] Anguera M. T. Blanco-Villaseñor A. Losada J. L. Sánchez-Algarra P. Onwuegbuzie A. J. (2018). Revisiting the difference between mixed methods and multimethods: is it all in the name? Qual. Quan. 52, 2757–70. 10.1007/s11135-018-0700-2

[B3] Anguera M. T. Camerino O. Castañer M. Sánchez-Algarra P. Onwuegbuzie A. J. (2017). The specificity of observational studies in physical activity and sports sciences: moving forward in mixed methods research and proposals for achieving quantitative and qualitative symmetry. Front. Psychol. 8:2196. 10.3389/fpsyg.2017.02196 29312061 PMC5742273

[B4] Anguera M. T. Portell P. Hernández-Mendo A. Sánchez-Algarra P. Jonsson G. K. (2021). “Diachronic analysis of qualitative data,” in Reviewer's Guide for Mixed Methods Research Analysis, eds. A. J. Onwuegbuzie and B. Johnson (Oxfordshire: Routledge), 125–138.

[B5] Bakeman R. Quera V. (2001). Using GSEQ with SPSS. Metodología de las Ciencias del Comportamiento 3, 195–214. 10.4236/psych.2015, 66080.29659707

[B6] Creswell J. W. Plano Clark V. L. (2007). Designing and conducting mixed methods research. Thousand Oaks, CA: SAGE Publications.

[B7] Hernández-Mendo A. Blanco-Villaseñor Á. Pastrana J. L. Morales-Sánchez V. Ramos-Pérez F. J. (2016). SAGT: Aplicación informática para análisis de generalizabilidad. Revista Iberoamericana de Psicología del Ejercicio y el Deporte 11, 77–89. Available online at: https://hdl.handle.net/2445/108553

[B8] Hernández-Mendo A. López J. A. Castellano J. Morales-Sánchez V. Pastrana J. L. (2012). Hoisan 1.2: software for use in observational methodology. Cuadernos de Psicología del Deporte 12, 55–77. 10.4321/S1578-84232012000100006

[B9] Magnusson M. S. (1996). Hidden real-time patterns in intra- and inter-individual behavior: description and detection. Eur. J. Psychol. Assess. 12, 112–123. 10.1027/1015-5759.12.2.11215939552

[B10] Magnusson M. S. (2020). T-pattern detection and analysis (TPA) with THEMETM: a mixed methods approach. Front. Psychol. 10:2663. 10.3389/fpsyg.2019.02663 31998165 PMC6965347

[B11] Magnusson M. S. Burgoon J. K. Casarrubea M. (Eds.). (2016). Discovering Hidden Temporal Patterns in Behavior and Interaction (Vol. 111). Berlin: Springer.

[B12] Soto-Fernández A. Camerino O. Iglesias X. Anguera M. T. Castañer M. (2022). LINCE PLUS software for systematic observational studies in sports and health. Behav. Res. Methods 54, 1263–1271. 10.3758/s13428-021-01642-1 34549384 PMC9170632

